# Functional miR-142a-3p Induces Apoptosis and Macrophage Polarization by Targeting *tnfaip2* and *glut3* in Grass Carp (*Ctenopharyngodon idella*)

**DOI:** 10.3389/fimmu.2021.633324

**Published:** 2021-06-28

**Authors:** Lizhu Tao, Yifan Pang, Anqi Wang, Lisen Li, Yubang Shen, Xiaoyan Xu, Jiale Li

**Affiliations:** ^1^ Key Laboratory of Freshwater Aquatic Genetic Resources Ministry of Agriculture and Rural Affairs, Shanghai Ocean University, Shanghai, China; ^2^ Institute of Fisheries of Chengdu Agriculture and Forestry Academy, Chengdu, China; ^3^ National Demonstration Center for Experimental Fisheries Science Education, Shanghai Ocean University, Shanghai, China; ^4^ Shanghai Engineering Research Center of Aquaculture, Shanghai Ocean University, Shanghai, China; ^5^ Comparative Endocrinology and Integrative Biology, Centre of Marine Sciences, Universidade Do Algarve, Faro, Portugal

**Keywords:** inflammation, apoptosis, macrophage, grass carp, miR-142a-3p

## Abstract

In the process of microbial invasion, the inflammation reaction is induced to eliminate the pathogen. However, un-controlled or un-resolved inflammation can lead to tissue damage and death of the host. MicroRNAs (miRNAs) are the signaling regulators that prevent the uncontrolled progress of an inflammatory response. Our previous work strongly indicated that miR-142a-3p is related to the immune regulation in grass carp. In the present study, we found that the expression of miR-142a-3p was down-regulated after infection by *Aeromonas hydrophila*. *tnfaip2* and *glut3* were confirmed as be the target genes of miR-142a-3p, which were confirmed by expression correlation analysis, gene overexpression, and dual luciferase reporter assay. The miR-142a-3p can reduce cell viability and stimulate cell apoptosis by targeting *tnfaip2* and *glut3*. In addition, miR-142a-3p also regulates macrophage polarization induced by *A. hydrophila*. Our results suggest that miR-142a-3p has multiple functions in host antibacterial immune response. Our research provides further understanding of the molecular mechanisms between miRNAs and their target genes, and provides a new insights for the development of pro-resolution strategies for the treatment of complex inflammatory diseases in fish.

## Introduction

MicroRNAs (miRNAs)—small, endogenous, non-coding RNAs approximately 22 bases in length—play a role in gene regulation by binding the 3ʹ non-coding regions of genes (1). miRNAs regulate various physiological processes, including disease (2), growth, and development (3, 4). miR-393a—the first reported miRNA—in *Arabidopsis thaliana* is involved in host resistance to bacterial infections (5). miRNAs are activated by invasive pathogenic bacteria, subsequently affecting a range of host cell functions such as immune response, cell cycle progression, cytoskeleton organization, and cell death or survival (6). In recent years, a large number of miRNAs are regarded as a fine-tuning regulator of immune responses that could involve in bacterial or viral infection by targeting immune-related genes in teleost fish (7–10). In Japanese flounder, pol-miR-novel-171 exerts a pro-apoptotic effect by down-regulating FAM49B to repressing apoptosis and affecting bacterial infection (11). The highly abundant miRNAs associated with macrophage differentiation and immune response in Atlantic salmon (12).

Apoptosis, a type of programmed cell death (13, 14), exerts a crucial function in many physiological processes, such as homeostasis maintenance, tissue and organ development, and immune defense (15, 16). Efficient clearance of apoptotic cells is a key process that can prevent inflammation and maintain self-tolerance under physiological conditions (17). Apoptotic cells are eliminated mainly by M2 macrophages (18), which promotes anti-inflammatory gene expression and thus contributes to inflammation resolution (19). Unlike M2 macrophages, M1 macrophages have pro-inflammatory and microbicidal functions (20). Kupffer cells, liver-resident macrophages that constitute 80–90% of all tissue macrophages in the body, are considered the first line of defense (21). However, studies on miRNAs regulating macrophage polarization have not been reported yet in teleost.

Our previous findings indicate that miR-142a-3p is differentially expressed in susceptible and disease-resistant grass carp kidney, suggesting that it is associated with immune regulation in lower vertebrates (22, 23). Over the years, many studies have underscored that miR-142 plays an important role in various physiological processes and in diseases that are characterized by a strong inflammatory response, such as sepsis (24). Sepsis is a severe disease characterized by systemic inflammatory response caused by invasive bacteria invading the blood (23, 25). Therapeutic perspectives targeting apoptosis and transforming macrophages into a proper phenotype could improve survival in sepsis (26, 27). Apoptosis (28) and macrophage polarization (29) are regulated by miRNAs and play key roles in immune diseases. In human intestinal epithelial cells, miR-4334, miR-219, and miR-338 attenuate lipopolysaccharide-induced apoptosis by inhibiting the TLR4-NF-κB-p53 pathway (30). Therefore, it is important to investigate the definitive role of miRNA-induced apoptosis and macrophage polarization in sepsis.

Grass carp (*Ctenopharyngodon idella*) is widely cultivated in China, especially in the headstream areas along the Yangtze and Pearl Rivers, with an annual production over 6 million tons (31). However, cultured grass carp are rather susceptible to various bacterial pathogens (32). In our previous study, we identified 21 miRNAs related to antibacterial immune processes in grass carp susceptible and resistant to *Aeromonas hydrophila* by combining target prediction with mRNA and miRNA expression patterns (23). In the present study, we aimed to further investigate the regulatory mechanism and function of grass carp miR-142a-3p and found that it regulated the apoptosis and macrophage polarization by targeting TNFα‐induced protein 2 (*tnfaip2*) and glucose transporter 3 (*glut3*).

## Materials and Methods

### Experimental Fish

Grass carp (average weight 750 g) were obtained from Binhai Farm of the Shanghai Ocean University, Shanghai, China. The fish allowed to acclimate in a disinfected rearing tank containing fully aerated water and with light conditions suitable for fish growth. The water temperature was maintained at 28 ± 2°C using a heater. The fish were raised in 400 L aerated tanks for two weeks before the experiment and fed twice daily (in the morning and late in the afternoon) at a ratio of 5% of the total biomass. All sampling tools were sterilized using 75% ethanol. After sampling, liver, heart, muscle, brain, skin, intestine, fin, kidney, gill, and spleen were immediately stored at -80°C. All the experimental fish (grass carp) were reared and handled according to the Guidelines on the Care and Use of Animals for Scientific Purposes by the Institutional Animal Care and Use Committee (IACUC) of Shanghai Ocean University, Shanghai, China. IACUC approved this study within the “Breeding of Grass Carp” project (approval number is SHOU-09-007).

### Culture and Infection of Grass Carp Kidney Cells

The grass carp kidney (CIK) cells were provided by the China Center for Type Culture Collection (Wuhan, China). The cells were cultured in M199 medium (Life Technologies, USA) supplemented with 10% heat-inactivated fetal bovine serum (FBS; Life Technologies) and penicillin-streptomycin solution (Gibco, USA) in a humidified incubator at 28°C under 5% CO_2_. Before the experiment, CIK cells were adjusted to 2 × 10^6^ cells/mL final concentration and incubated in 6-well or 24-well culture plate for 24 h. Next, the cells were washed with phosphate-buffered saline (PBS), and 1 mL of antibiotic-free nutrient solution (M199 with 10% FBS) and 100 µL *A. hydrophila* (10^3^ CFU/mL) were added to the well. CIK cells treated with PBS were used as control. Cells collected at different time points post infection (0, 12, 24, 36 h) were used for analysis by real-time quantitative PCR (33, 34). All samplings were performed in triplicates.

### Grass Carp Kupffer Cell Isolation and Primary Cell Culture

Grass carp liver macrophages were prepared by discontinuous density gradient centrifugation (35). Briefly, grass carp liver was dissected with sterilized scissors and tweezers. All tissues were washed thrice in PBS containing 1% penicillin-streptomycin solution to eliminate contamination. Then, tissues were homogenized with a syringe and filtered using 70-mesh cell filter. The tissue filtrate was added dropwise to the 51% Percoll solution ensuring that the interface was not broken. The samples were centrifuged at 4°C for 30 min. The white liquid in the middle layer was collected, re-suspended, and centrifuged at 4°C for 10 min; the supernatant was discarded. The Kupffer cells collected were seeded in 6-well plate and cultured at 28°C for 6 h. Non-adherent cells were removed, and adherent cells were incubated in complete medium (L-15, 10% FBS, and 1% penicillin and streptomycin) at 28°C under 5% CO_2_.

### miR-142a-3p Target Prediction

The miR-142a-3p binding sites were predicted using miRanda and RNAhybrid algorithms (36, 37). RNAhybrid predicts secondary structures between the miRNA and the target gene through Minimum Free Energy (MFE) calculations. miRanda algorithm was assessing the thermodynamic folding energy of a miRNA and binding sites duplex.

### Recombinant Plasmid Construction

The 3′ UTRs of *tnfaip2* or *glut3* fragments containing presumptive miR-142a-3p binding sites were amplified by PCR. The amplicon was cloned into the pmirGLO vector (Promega Wisconsin, USA) to generate the recombinant plasmid pmirGLO-*tnfaip2* or pmirGLO-*glut3*. All constructed plasmids were verified by Sanger sequencing (Sangon Biotech, China) and extracted using the Endotoxin-Free Plasmid DNA Miniprep kit (Tiangen, China) for further use in the luciferase reporter assay.

### RNA Extraction and cDNA Synthesis

Total RNA was extracted using TRIzol reagent from Kupffer cells or CIK cells. RNA concentration was measured using Nanodrop 2000 (Thermo Fisher Scientific), and RNA integrity was visualized using 1% agarose gel electrophoresis. 1 µg of total RNA was reverse transcribed using a PrimeScript RT reagent kit with gDNA Eraser (TaKaRa, Dalian, China), cDNA synthesis was conducted using oligo-dT ligated to specific 5ʹ sequence. For miRNA, cDNA was prepared using aMir-X miRNA First Strand Synthesis kit (Clontech, Palo Alto, USA). This kit adds poly (A) to the 3′ end of the miRNAs and performs reverse transcription.

### Real-Time PCR

Quantitative reverse transcription PCR (qRT-PCR) analysis was performed on CFX96 (Bio-Rad Laboratories, Hercules, CA, USA) using TB Green Advantage qPCR Premix (Clontech). qPCR was performed in a final reaction volume of 25 µL; each reaction mixture included 9.5 µL ddH_2_O, 12.5 µL of TB Green Advantage Premix, 0.5 µL forward primer (10 µM), 0.5 µL reverse primer for mRNA or mRQ 3ʹ primer (Clontech) for miRNA (10 µM), and 2 µL of cDNA. The following qPCR cycling conditions were used: 95°C for 10 s and 40 cycles at 95°C for 5 s and 60°C for 20 s. Dissociation curve analysis was performed after each assay to determine target specificity. miR-101 (38) and *β-actin* (39, 40) were used to normalize the relative expression of miRNA and mRNA, respectively. The primers used for qPCR are listed in **Table 1**. Each experiment was performed in quadruplicate.

**Table 1:** Primer sequences used in the present study.

Gene Name	Primer sequence		Application
	Forward primer (5ʹ-3ʹ)	Reverse primer (5ʹ-3ʹ)	
***miR-142a-3p***	TGTAGTGTTTCCTACTTTATGGA	mRQ 3’ primer	qPCR
***miR-101a***	TACAGTACTGTGATAACTGAAG	mRQ 3’ primer	qPCR
***tnfaip2***	TCAACTGTGTTCCACCATTC	GTTATCCACCCACTTCCTCT	qPCR
***pmirGLO-tnfaip2***	GAGCTCGGGCTACAGTTGGAGCAATGG	TCTAGAGGTATAACAGAACGGGCGAGG	Vector construction
***glut3***	CTGGTCGGCTGATTATTGGG	AGGATGGCTGGTAGCACGGT	qPCR
***pmirGLO-glut3***	GAGCTCGCTGGTCGGCTGATTATTGGG	TCTAGAGAGGATGGCTGGTAGCACGGT	Vector construction
***tnf-α***	ACCCTGAAGTCTCTAATAAAACCC	GTGGCTCATATGCACAATGTCT	qPCR
***il-1β***	TCTCCTCGTCTGCTGGGTGT	CAAGACCAGGTGAGGGGAAG	qPCR
***il-6***	ATTAGAAACTGAACGGCGAG	TCTGAATGTTGTGCTGGGAT	qPCR
***il-8***	TCTACCCTCCTAGCCCTCACTG	TCATGGTGCTTTGTTGGCAAGGA	qPCR
***il-10***	ACGAGAACGTGCAACAGA	TGGCAAACTCAAAGGGAT	qPCR
***tgf-β***	TTACGGCTTCGGATTAAG	TGGCAGTGTCACCTCTCT	qPCR
***β-actin***	CATGCCATCCTCCGTCTGGA	GGATGGCTGGAACAGAGCCT	qPCR

### Agomir and Antagomir

miR-142a-3p agomir and the antagomir were commercially synthesized by GenePharma (Shanghai, China). Agomir is a specially labeled and chemically modified double-stranded small RNA that modulates the biological function of target genes by mimicking endogenous miRNAs. Antagomir is a specially designed and chemically modified single-stranded small RNA based on the microRNA mature sequence, which is a potent blocker for the inhibition of endogenous microRNAs (41).

### Effect of miR-142a-3p on *tnfaip2* or *glut3*

CIK cells were seeded in 24-well plates with 80% cell density, miR-142a-3p agomir, antagomir or native control were separately introduced into CIK cells at a final concentration of 100 nM by transfection using Lipofectamine 3000 reagent (Invitrogen). At 48 h post-transfection, the cells were lysed with TRIzol, RNA extraction and qRT-PCR were performed as described above.

### Transfection and Dual Luciferase Reporter Detection

For the transfection experiment, CIK cells (2 × 10^6^ cells/mL) from 24-well plate were cultured in M199 medium containing 10% FBS and 1% penicillin-streptomycin solution with 5% CO_2_ at 37°C. After 24 h, co-transfection was performed with the pmirGLO-*tnfaip2* or pmirGLO-*glut3* vector (100 ng), miR-142a-3p agomir or negative control (100 nM) using the Lipofectamine 3000 reagent. into CIK cells. After 48 h of transfection, firefly and Renilla luciferase activity was measured using dual luciferase reporter assay (Promega). Firefly luciferase activity was normalized to Renilla luciferase activity. For each experiment, three independent experiments were conducted, and each experiment was done in triplicate.

### Caspase 3/7 Activity and Cell Viability Assessment

Apoptosis of CIK cells was detected using a Caspase-Glo3/7 assay (Promega, USA) according to the manufacturer’s instructions. In brief, CIK cells were first transfected with miR‐142a-3p agomir or antagomir (100 nM) for 48 h. Subsequently, the cells were incubated with Caspase-Glo 3/7 reagent at room temperature for 2 h. Caspase 3/7 activity was measured using GloMax Multi Jr Single Tube Detection System (Promega, USA). Cell Counting Kit-8 (CCK8) reagent (10 µL) (Beyotime, Shanghai, China) was added into each well, and the cells were incubated for 2.5 h. The detailed protocol of calculating relative cell viability was described in a previous study. Briefly, each time point relative cell viability (%) related to the control wells was calculated by the following formula: Cell viability (%) = (Treated group A450 nm - Blank control A450 nm)/(Negative control A450 nm - Blank control A450 nm) × 100% (42).

### Statistical Analysis

Relative gene expression was calculated using the 2^−ΔΔCT^ method. All qPCR data was log-transformation before statistical test. Significant differences between groups were determined by one-way analysis of variance (ANOVA) followed with Duncan multiple comparison test or two-tailed Student’s t-test. All data are described as the mean ± standard deviation (SD) of three independent experiments. Differences in mean values were considered statistically significant when *p* < 0.05 and extremely significant when *p* < 0.01.

## Results

### MiR-142a-3p Is Involved in Bacterial Infection

**Figure 1:**
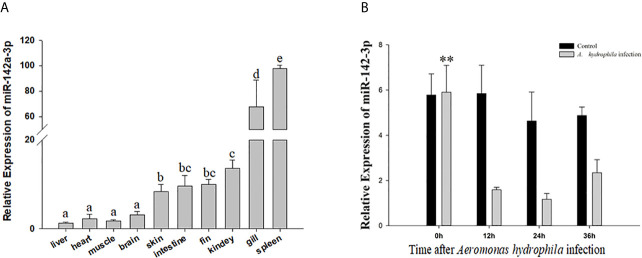
Analysis of miR-142a-3p expression by qRT-PCR **(A)** in 10 tissues of grass carp **(B)** in grass carp kidney (CIK) cells 0, 12, 24, and 36 h after *A. hydrophila* infection. All values represent the mean ± SD of three independent experiments. Different lowercase letters indicate statistically significant differences (p < 0.05). Asterisks indicate significant differences (**p < 0.01).

*In vivo*, miR-142a-3p was found to be expressed in all tested tissues and especially highly expressed in gill and spleen (**Figure 1A**). *In vitro*, we further showed that miR-142a-3p was downregulated in CIK cells after *A. hydrophila* infection at 12 h, 24 h, and 36 h (**Figure 1B**).

### Prediction and Validation of miR-142a-3p Target Genes

**Figure 2:**
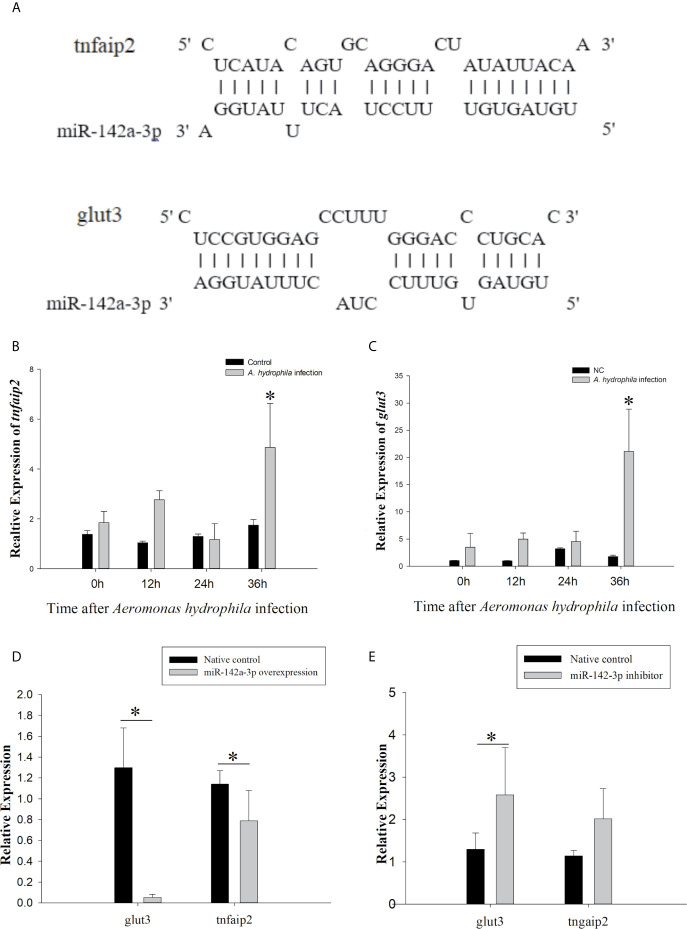
Prediction and validation of target genes of miR-142a-3p. **(A)** Binding site (as shown with a solid line) of miR-142a-3p to 3′ UTR of *tnfaip2* and *glut3.* **(B, C)** Expression profiles of *tnfaip2* and *glut3* in grass carp kidney (CIK) cells at 0, 12, 24, and 36 h following *A. hydrophila* infection. **(D, E)** Relative expression of *tnfaip2* and *glut3* in CIK cells after miR-142a-3p overexpression or inhibition. All values represent the mean ± SD of three independent experiments. Asterisks indicate significant differences (*p < 0.05).

**Figure 3:**
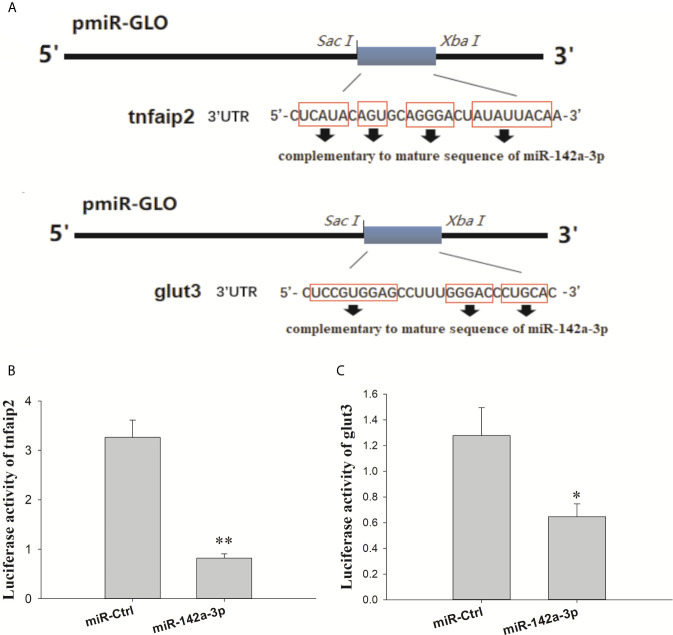
**(A)** Schematic diagram of the SacI/Xbal sites. **(B, C)** CIK cells were transfected with miR-142a-3p agomir or control solution, along with the recombinant plasmids pmirGLO-*tnfaip2* or pmirGLO-*glut3* for 48 h, and the luciferase activity was determined. All values represent the mean ± SD of three independent experiments. Asterisks indicate significant differences *p < 0.05, **p < 0.01.

The putative binding site for miR-142a-3p was predicted to be on the 3’ UTR of *tnfaip2* and *glut3* (**Figure 2A**). The MFE (minimum free energy) of the binding between miR-142a-3p and *tnfaip2* is -22.9 kcal/mol, and that of the binding between miR-142a-3p and *glut3* is -23.9 kcal/mol. In CIK cells infected with *A. hydrophila*, the expression patterns of miR-142a-3p and target genes (*tnfaip2* and *glut3*) were found to be negatively correlated. After bacterial infection, miR-142a-3p expression was downregulated (**Figure 1B**), whereas *tnfaip2* (**Figure 2B**) and *glut3* (**Figure 2C**) expression was significantly upregulated. *Tnfaip2* and *glut3* expression was inhibited by miR-142a-3p (**Figure 2D**), and it increased when miR-142a-3p was inhibited (**Figure 2E**). The direct interactions between miR-142a-3p with *tnfaip2* and *glut3* were confirmed using the dual luciferase reporter system (**Figure 3A**). The luciferase activity of pmirGLO-*tnfaip2* or pmirGLO-*glut3* was significantly reduced by miR-142a-3p in CIK cells (**Figures 3B, C**). Overall, the above data fully demonstrate that *tnfaip2* and *glut3* are the target genes of miR-142a-3p.

### miR-142a-3p Promotes Apoptosis

**Figure 4:**
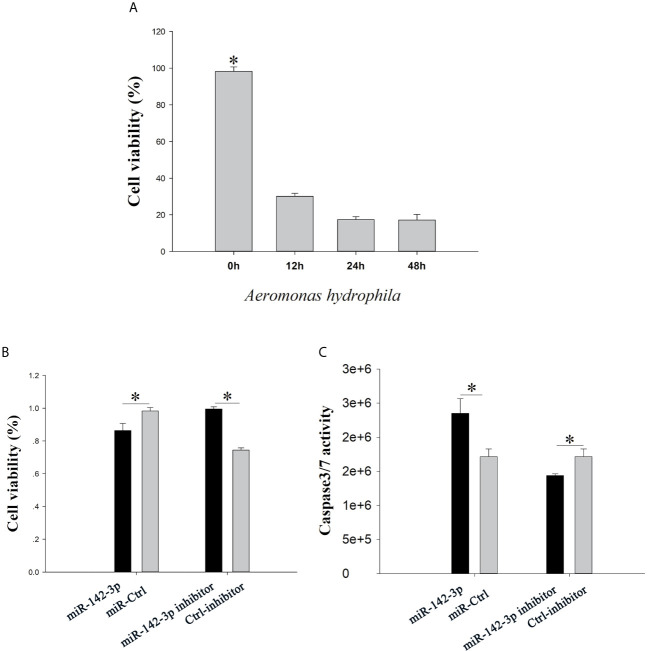
**(A)** grass carp kidney (CIK) cells were treated with *A. hydrophila*, the cell counting kit-8 (CCK8) assay was used to evaluate cell viability. **(B)** CIK cells were transfected with miR-142a-3p agomir or antagomir, and the CCK8 assay was used to evaluate cell viability. **(C)** The caspase-Glo 3/7 kit was used to detect the caspase 3/7 activity. All values represent the mean ± SD of three independent experiments. *p < 0.05.

The CCK8 assay showed that CIK cell viability reduced in a time-dependent manner (**Figure 4A**). Overexpression of miR‐142a-3p stimulated cell death, whereas inhibition of miR-142a-3p enhanced the cell survival rate (**Figure 4B**). In addition, measurement of caspase 3/7 enzyme activity showed that inhibition and overexpression of miR-142a-3p reduced and increased the cell apoptosis rate, respectively (**Figure 4C**).

### MiR-142a-3p Modulate Kupffer Cell Polarization

**Figure 5:**
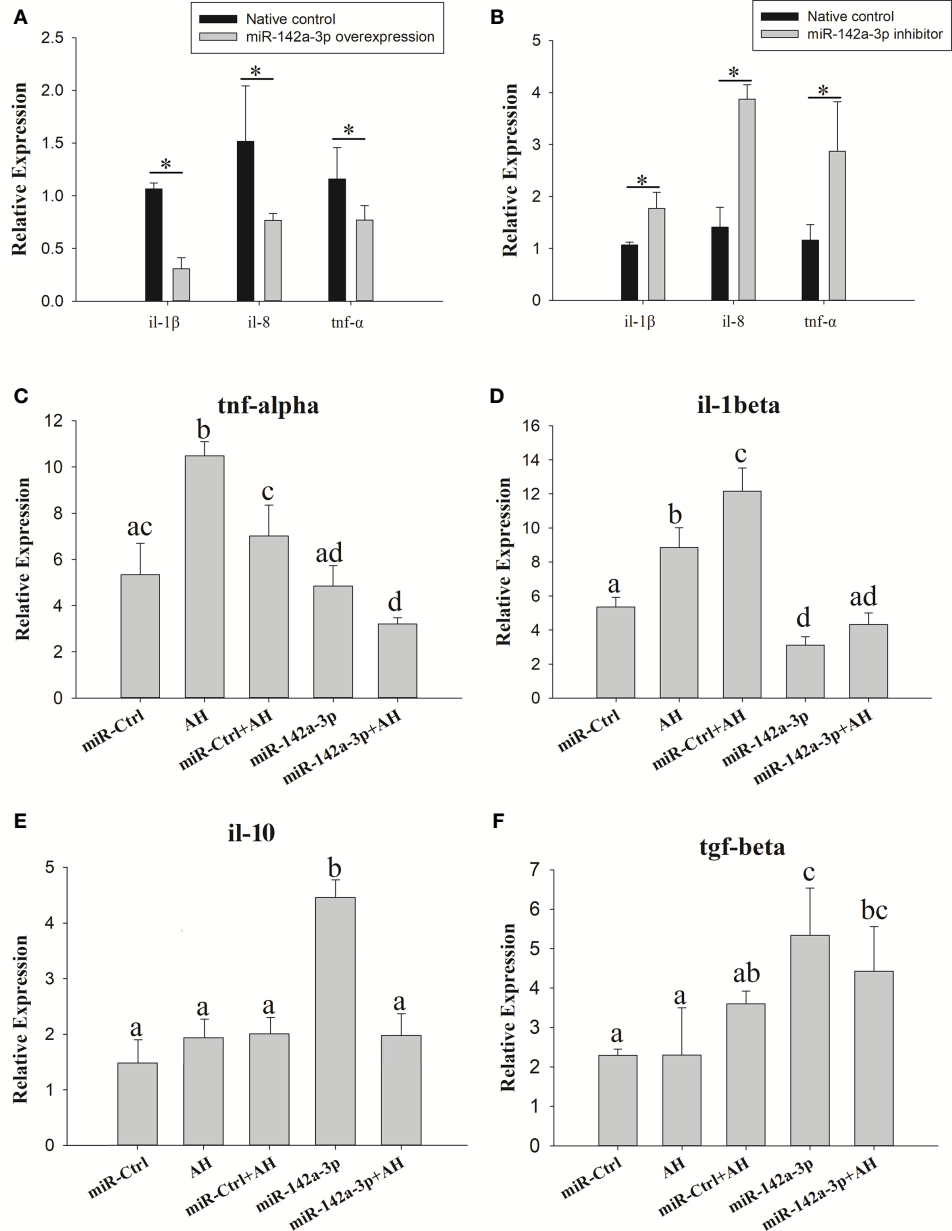
Grass carp cells (CIK) were transfected with either the miR-142a-3p agomir **(A)** or antagomir **(B)**. After 48 h, the level of tnf-α, il-1β, and il-8 expression was determined using qPCR. Kupffer cells were transfected with miR-142a-3p agomir or control solution, and then the Kupffer cells were infected with Aeromonas hydrophila. The mRNA levels of il-1β, tnf-α, il-10, and tgf-β **(C–F)** were analyzed by qRT-PCR 6 h after infection. All values represent the mean ± SD of three independent experiments. Different lowercase letters indicate statistically significant differences (*p* < 0.05). Asterisks indicate significant differences (*p < 0.05). AH, *A. hydrophila*.

The expression of downstream inflammatory factors (*tnf-α, il-1β*, and *il-8*) was evaluated to further determine whether miR-142a-3p affects the inflammatory response. The results showed that *tnf-α, il-1β*, and *il-8* were significantly downregulated in the miR-142a-3p overexpression group (**Figure 5A**). In contrast, their expression was significantly upregulated in the miR-142a-3p inhibition group (**Figure 5B**).

We also found that *A. hydrophila* can stimulate the expressions of pro-inflammatory factors *tnf-α* and *il-1β*. Expression of *tnf-α* and *il-1β* was decreased when miR-142a-3p was overexpressed in Kupffer cells (**Figures 5C, D**). miR-142a-3p significantly enhanced the expressions of anti-inflammatory factors *il-10* and *tgf-β* in Kupffer cells (**Figures 5E, F**).

## Discussion

Pathogens such as viruses, bacteria, fungi, and other environmental stimuli are latent stressors that damage animal tissues and cells and trigger inflammation (43). Alleviation and prevention of inflammation is crucial for survival (44); uncontrolled inflammation is associated with several diseases, for which new therapeutic interventions need to be developed (45). Uncontrolled inflammation results from not only persistent activation of inflammatory signals but also a lack of clearance of dead cells and inhibition of pro-inflammatory cytokines (46). Previous studies suggest that miRNAs mediate inflammation by regulating certain genes and perform multiple functions during this process (47). For instance, miR-21 regulates NF-κB activity, thus transforming activated macrophages into cells with a reparative function (48), promotes the expression of inflammatory factors by downregulating TIMP3 (49), and inhibits T cell apoptosis by targeting tumor suppressors (50). mir-142a-3p was one of 21 differentially expressed miRNAs between grass carp susceptible and resistant to *A. hydrophila* (23, 51); the findings were consistent with a previous report that mir-142a-5p was involved in the regulation of the IL-6 signaling pathway, thereby modulating inflammation (52). In the present study, we focused on the function of miR-142a-3p in the inflammatory response of grass carp infected with *A. hydrophila*.

The expression levels of miRNAs varied greatly according to tissues and were highly function skewed (53). We found that miR-142a-3p was highly expressed in immune organs such as the trunk kidney, gill, and spleen, and its expression was significantly altered in CIK cells infected with *A. hydrophila*. Trunk kidney served as an important organ, and played a vital role to trigger innate immune responses in grass carp (54). The interbranchial lymphoid tissue most likely is a secondary lymphoid structure (55), and the spleen also serves as a secondary lymphoid organ. It suggested that the miR-142a-3p played a vital role to regulation innate immune responses in grass carp. For functional validation experiments, we focused on *tnfaip2* and *glut3* because they were predicted to be the potential targets of miR-142a-3p. Expression correlation analysis, gene overexpression, and dual luciferase reporter assay suggested that *tnfaip2* and *glut3* are directly targeted by miR-142a-3p. TNFAIP participates in several biological processes, including cell proliferation, apoptosis, and inflammatory response (56). It acts as a negative regulator of NF-κB activity and is associated with the high mortality in patients with sepsis (57). The Glut family comprises 14 members that transport glucose or other substrates in different tissues. Glut3 shows high affinity and activity (58) and is involved in multiple pathways, including cAMP, NF-kB, and p53 signaling pathways (59). GLUT3 inhibition induces apoptosis in HeLa cells (60), and downregulation of GLUT3 with siRNA promotes apoptosis in acute myeloid leukemia cells (61). Furthermore, our study validated previously reported results that GLUT3 inhibition promotes cell apoptosis (62). There is a body of evidence supporting that a single miRNA regulates many genes involved in the same biological process. The results of the CCK8 and caspase 3/7 assay in the present study suggest that miR-142a-3p downregulation reduced apoptosis and promoted cell survival, whereas miR-142a-3p overexpression showed the opposite results. Furthermore, our study provides insights into the miRNA-mediated regulatory mechanism of cell survival during the interaction between the host and pathogenic bacteria *in vitro* (23).

Macrophages are vital innate immune cells found in almost all tissues (63) and contribute to a broad spectrum of pathologies. Activation of mononuclear phagocytes, depending on the type of extracellular stimulants, leads to cell polarization, producing either M1 or M2 macrophages through the classical or non-classical activation pathway, respectively (64). The expression of inflammatory factors is often closely associated with macrophage polarization. Cells in the M1 pathway play a pro-inflammatory role and secrete pro-inflammatory cytokines such as *iL-1β* and *tnf-α*, which inhibit cell proliferation and antigen presentation (65, 66). The activation of M2 pathway has anti-inflammatory functions in parasitic infections, mainly reflected by enhanced cell proliferation, tissue repair and reconstruction, tumor formation, and recovery of inflammatory responses (67). A previous study suggests that mannose-binding lectin significantly enhances *il-10* and *tgf-β* expression in M2-phenotype macrophages in *A. hydrophila*-infected grass carp (68). However, it has been shown that steatotic livers fail to significantly upregulate *il-10* expression in response to injury (69). Our results show that the il-10 no significant effects after *A. hydrophila*. It may indirectly reflect the steatotic liver of grass carp in this study. Many miRNAs have been found to participate in macrophage polarization (29). For example, overexpression of miR-125a-5p inhibited LPS-induced M1 marker expression while enhancing the expression of IL-4-induced M2 markers (70). Moreover, miR-221 targets *tnfaip2* to reduce the inflammatory response of neuronal cells during spinal cord ischemia-reperfusion (71). In the present study, the expression profiles of cytokines (*il-1β*, *tnf-α*, *il-10*, and *tgf-β*) indicated that miR-142a-3p regulates the biological function of macrophages by affecting macrophage polarization. Collectively, these findings in grass carp expand the knowledge of the immune regulatory networks of miRNA in teleost fish.

Growing evidence has been shown that the innate immune system can be regulated by miRNAs (72). However, the mechanism underlying miRNA-mediated simultaneous activation of multiple immune pathways remains unknown (73). Macrophage encounter, interaction with, and uptake of apoptotic cells during inflammation resolution (74). In the presence of bacterial or LPS, the clearance or induces of apoptotic cells (efferocytosis) with macrophage deviation toward an anti-inflammatory phenotype is relevant (75). In this study, miR-142a-3p could simultaneously trigger macrophage polarization and apoptosis through the synchronous regulation of the expression of grass carp genes *tnfaip2* and *glut3*. These observations indicate that the intervention of key miRNAs may potentially alleviate the genes that involved in the related physiological function.

In summary, we concluded that miR-142a-3p regulates the cell viability and promotes apoptosis by targeting *tnfaip2* and *glut3*. miR-142a-3p also regulates macrophage polarization induced by *A. hydrophila*. Therefore, our results provide critical insights into the immunogenic role of miR-142a-3p in teleost and provide further evidence that miRNAs play an important role in the immune response of teleost infected with bacteria. However, the precise mechanisms associated with the role of miR-142a-3p require further research.

## Data Availability Statement

The datasets presented in this study can be found in online repositories. The names of the repository/repositories and accession number(s) can be found in the article/**Supplementary Material**.

## Ethics Statement

The animal study was reviewed and approved by Institutional Animal Care and Use Committee (IACUC) of Shanghai Ocean University.

## Author Contributions

LT, XX, and JL conceived and designed the experiments. LT performed the majority of the experiments, with the help of YP, AW, and YS. LT and XX analyzed the data. LT wrote the first draft of the manuscript under the supervision of XX. XX contributed to manuscript revision, read, and approved the final version. All authors contributed to the article and approved the submitted version.

## Funding

Financial support was received from National Natural Science Foundation of China Youth Project (Grant No. 31802285), China Agriculture Research System of MOF and MARA (CARS-45-03), and Special fund for science and technology development of Shanghai Ocean University.

## Conflict of Interest

The authors declare that the research was conducted in the absence of any commercial or financial relationships that could be construed as a potential conflict of interest.

## Supplementary Material

Supplementary Figure 1 Kupffer cells (arrows) were isolated from healthy grass carp liver.
