# Supplementary figures and images for: Functional miR-142a-3p Induces Apoptosis and Macrophage Polarization by Targeting tnfaip2 and glut3 in Grass Carp (Ctenopharyngodon idella)
Source: Front Immunol. 2021 Jun 28;12:633324. doi: 10.3389/fimmu.2021.633324 (PMC8273434; doi:10.3389/fimmu.2021.633324)

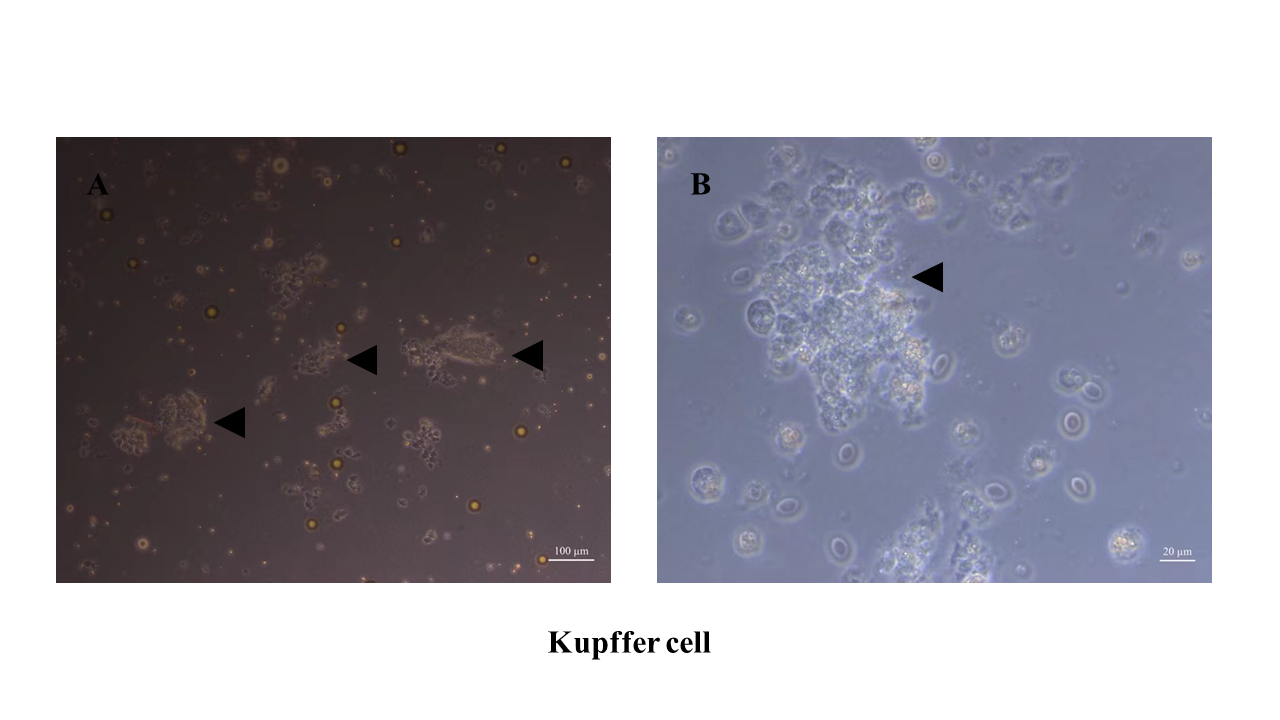

Supplement: Supplementary Figure 1 — Kupffer cells (arrows) were isolated from healthy grass carp liver. [file Image_1.tif]
